# Biphasic low-grade nasopharyngeal papillary adenocarcinoma: a case report and literature review

**DOI:** 10.1186/s12907-018-0076-1

**Published:** 2018-10-04

**Authors:** Hidenori Yokoi, Yuichi Terado, Masachika Fujiwara, Yuma Matsumoto, Tetsuya Ikeda, Koichiro Saito

**Affiliations:** 1 Department of Otolaryngology, Head and Neck Surgery, Kyorin University School of Medicine, 6-20-2 Shinkawa, Mitaka, Tokyo 181-8611 Japan; 2 Department of Pathology, Kyorin University School of Medicine, 6-20-2 Shinkawa, Mitaka, Tokyo 181-8611 Japan

**Keywords:** Biphasic tumor, Immunohistochemistry, Nasopharyngeal papillary adenocarcinoma, Spindle cell component, Thyroid transcription factor-1

## Abstract

**Background:**

Low-grade nasopharyngeal papillary adenocarcinoma (LGNPPA) is distinctly rare. We report a patient with a uniquely biphasic LGNPPA; additionally, we review similar tumors reported in the literature.

**Case presentation:**

A 56-year-old man presented with an asymptomatic pedunculated tumor in the vault of the nasopharynx, at the junction of the nasal septum and the roof, which was discovered during screening for laryngeal cancer. To obtain a definitive diagnosis, the patient underwent endoscopic endonasal surgery under general anesthesia. Immunohistochemical analysis of the tumor revealed it to be an LGNPPA with a prominent spindle cell component.

**Conclusion:**

To our knowledge, this is the fourth reported LGNPPA exhibiting a spindle cell component and the second with a prominent pathological condition. The prognosis of LGNPPA is usually excellent. Therefore, it is important for clinicians to scrutinize the lesion’s pathology to avoid unnecessary, disfiguring surgery.

## Background

Low-grade nasopharyngeal papillary adenocarcinoma (LGNPPA) is rare, and was first described and characterized by Wenig et al. in 1988 [1]. These tumors were considered a separate entity from other adenocarcinomas, including papillary adenocarcinomas of the sinonasal area [1]. Other patients reported since then include two described by Carrizo and Luna in 2005 who had LGNPPA that exhibited positive immunostaining for thyroid transcription factor-1 (TTF-1) [2]. The term “thyroid-like nasopharyngeal papillary adenocarcinoma (TL-NPPAC)” was coined based on the tumor’s immunohistochemical features and histological characteristics that include a papillary structure, psammoma bodies, neoplastic cells with overlapping nuclei, and clear chromatin [2]. Approximately 18 patients with TL-NPPAC have been reported to date in the English literature [3–5]. Petersson et al. recently reported a patient with an LGNPPA exhibiting a prominent spindle cell component; they referred to the tumor as a “biphasic low-grade nasopharyngeal papillary adenocarcinoma” [6].

Here, we report a second patient with asymptomatic LGNPPA that was positive for TTF-1 and showed biphasic neoplasm characteristics that included a papillary structure as well as prominent spindle cells; this tumor was incidentally discovered in a middle-aged man during a medical examination. We also review the literature as it relates to our patient’s clinical presentation, treatment progression, and pathological features.

## Case presentation

**Fig. 1:**
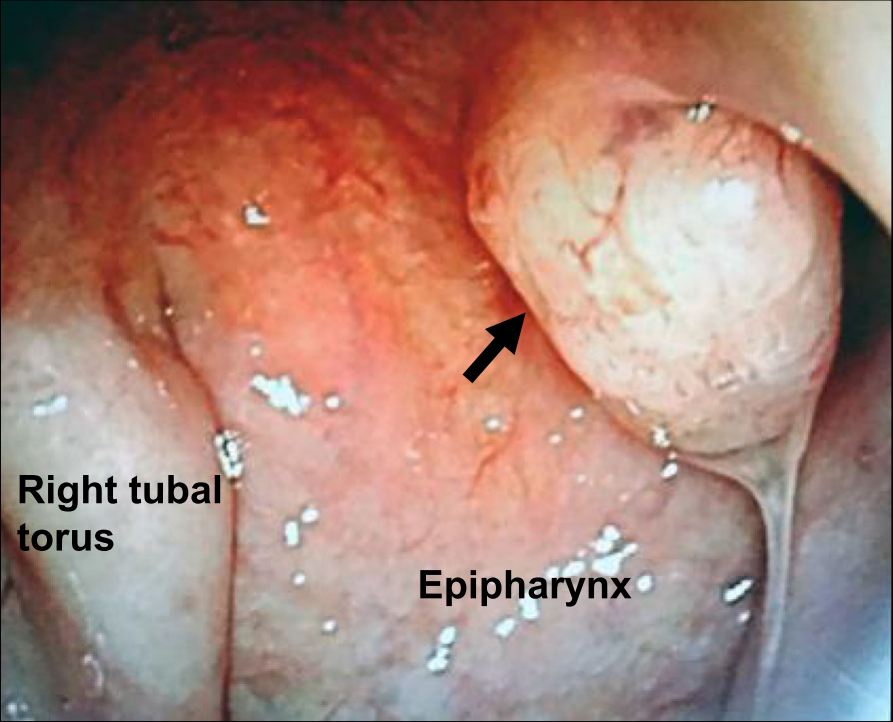
Local findings on electron spectroscopy (right channel view). The tumor is indicated by the black arrow

A round tumor with a diameter of approximately 10 mm that involved the posterior end of the nasal septal mucosa at the midline of the epipharynx was discovered in a 58-year-old man while screening for laryngeal cancer (Fig. 1). The patient was referred to our department for further evaluation, whereupon imaging analyses and a regional biopsy were performed under local anesthesia using a biopsy fiberscope. Pathological findings resembled an inverted ductal papilloma of the salivary glands, but did not produce a definitive diagnosis. The patient had a history of renal cancer for which he had undergone surgery 5 years prior.

**Fig. 2:**
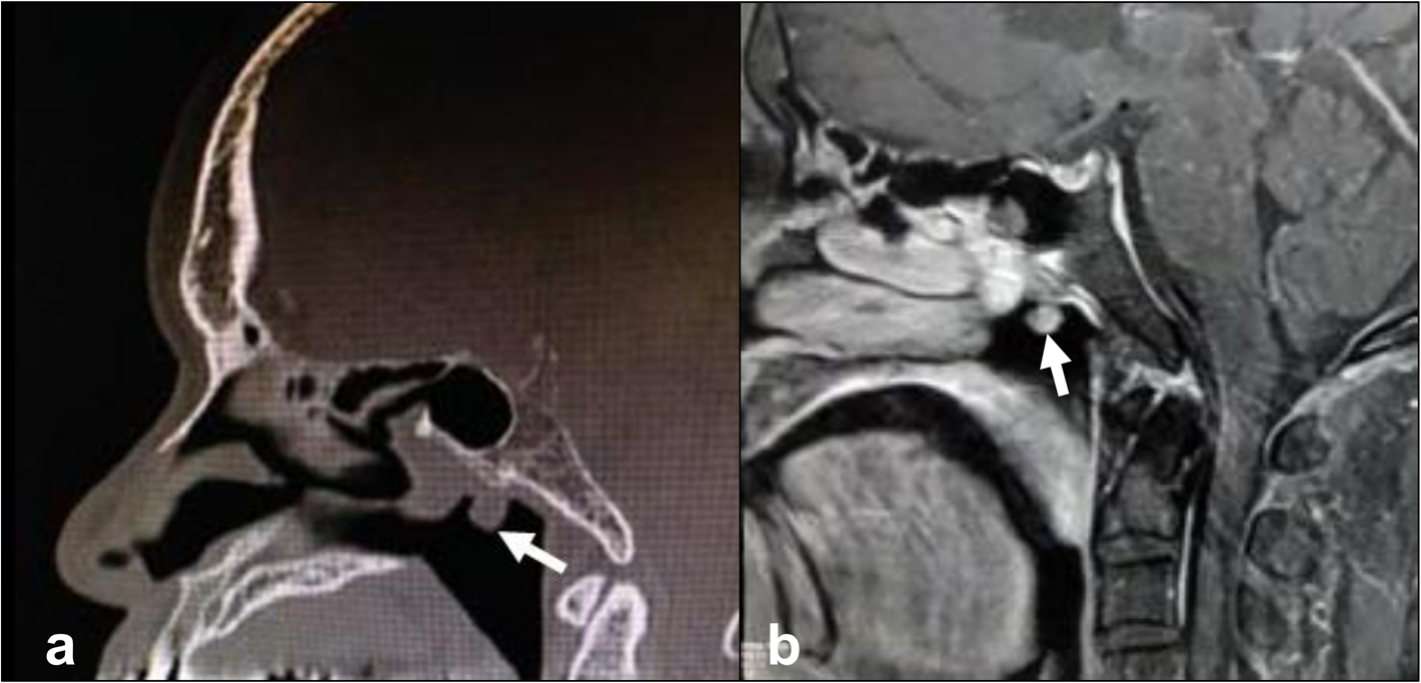
Tumor imaging. **a** Computed tomography image (no enhancement) and (**b**) magnetic resonance image (T1-weighted image gadolinium positive). White arrows show the tumor

Plain computed tomography (CT) revealed a soft tissue shadow tumor approximately 10 mm in size in the vault of the nasopharynx at the junction of the nasal septum and roof (Fig. 2a). Magnetic resonance imaging (MRI) showed a 10 mm mass at the same location as that observed on CT. T1- and T2-weighted images showed the same intensities as that of the nasal concha, and a regular contrast effect was observed (Fig. 2b). These MRIs suggested a benign tumor. Furthermore, positron emission tomography did not show any abnormal uptake of ^18^F-fludeoxyglucose in the nasopharynx, thyroid gland, or elsewhere in the body.

To excise the tumor and obtain a definitive pathological diagnosis, surgery was performed under general anesthesia using an endoscopic endonasal approach. First, an electrocautery needle was used for electrocoagulation and excision. Next, a suction curette was used for exfoliation of the tumor. Finally, the suction probe of the electrocautery device was used for electrocoagulation to stanch the bleeding.

**Fig. 3:**
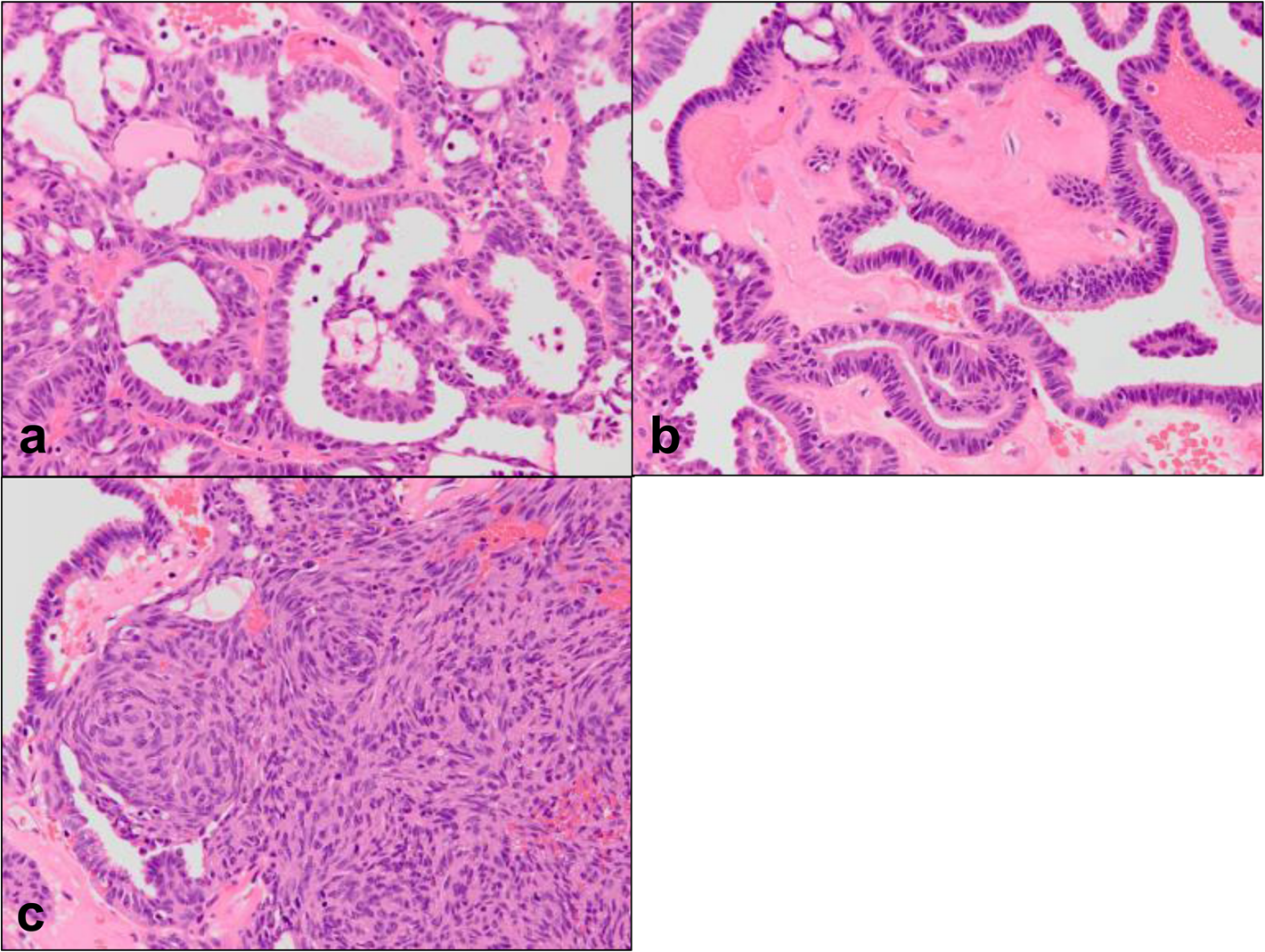
Hematoxylin-eosin staining of tumor samples. **a** Tubular formation, (**b**) papillary structure, and (**c**) solid growth of spindle cells. All magnifications are × 200

**Fig. 4:**
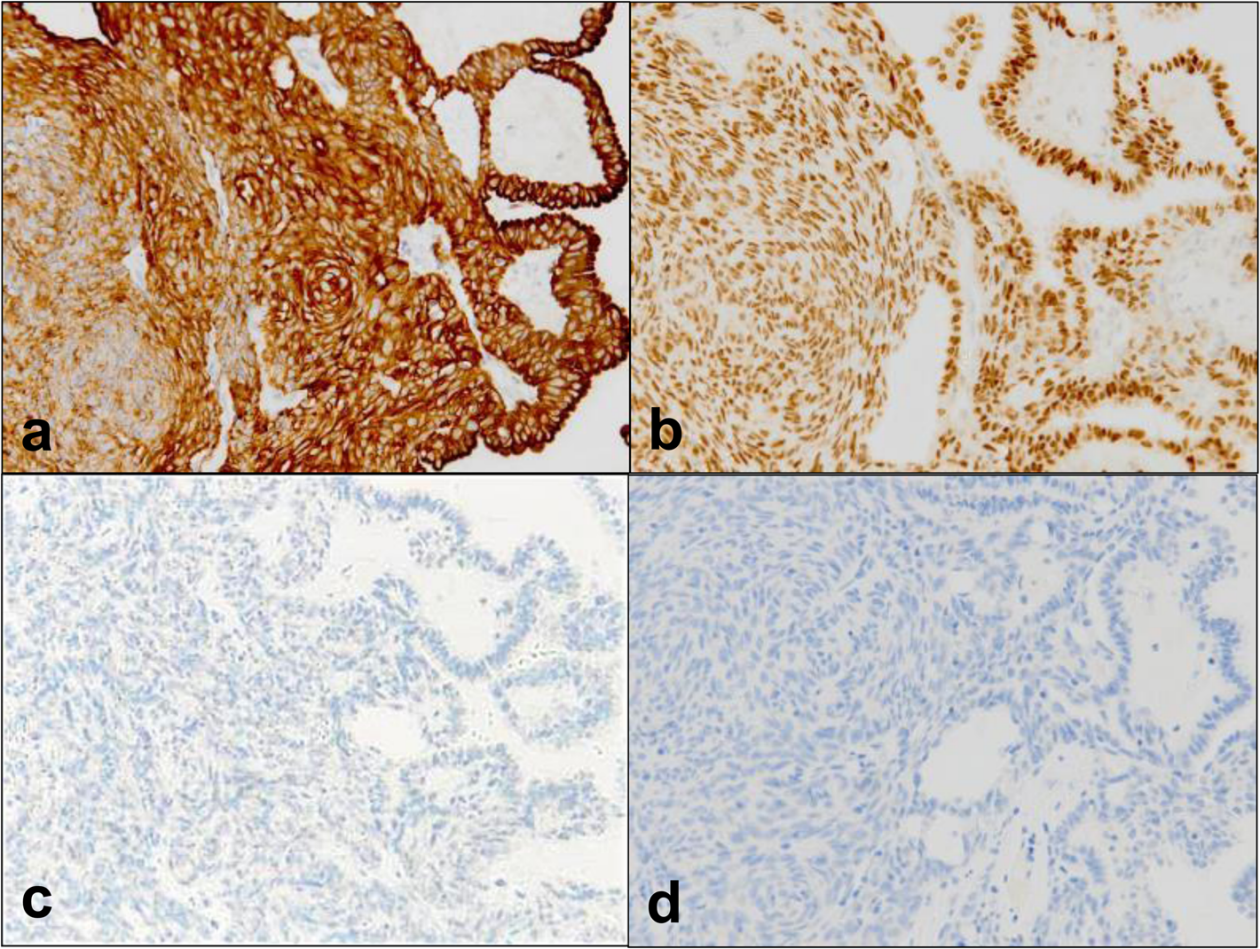
Immunohistochemical staining of tumor samples. The left side shows spindle cells while the right side shows columnar cells. The tumor was positive for (**a**) CK7 and (**b**) thyroid transcription factor-1, and was negative for (**c**) PAX8 and (**d**) p40. All magnifications are × 200

Hematoxylin-eosin staining showed that the tumor had a papillary structure lined by a columnar epithelium with a hyalinized fibrous core, and was additionally composed of sheets of spindle cells (Fig. 3); these two types of structures merged imperceptibly. A negative tumor margin was confirmed after surgery, and immunohistochemical studies showed that both columnar and spindle cells were diffusely positive for CK7 (Fig. 4a), TTF-1 (Fig. 4b), CK19, and vimentin (data not shown); however, they were negative for CK20, p63, smooth muscle actin (SMA), S-100, Epstein-Barr-encoded RNA (EBER), p16, human papillomavirus (HPV), and thyroglobulin (data not shown). The Ki-67 index was approximately 2–3%. P53 was irregularly positive in a small number of cells, suggesting wild-type status.

We diagnosed the tumor as a biphasic LGNPPA with a prominent spindle cell component [6]. No postoperative adjuvant treatment was administered. The patient is well and free of disease 34 months after the surgery.

## Discussion

Primary nasopharyngeal adenocarcinoma is a rare malignancy that accounts for ≤5% of all nasopharyngeal cancers [7, 8]. This tumor type has been categorized widely into two subtypes: the conventional/surface origin type and the salivary gland type [7, 8]. The former is usually a low-grade tumor with papillary configuration and likely originates from the nasopharyngeal surface mucosa, whereas the latter includes epithelial-myoepithelial carcinomas, mucoepidermoid adenocarcinomas, adenoid cystic carcinomas, and polymorphous low-grade adenocarcinomas [9]. Based on its low grade and site of occurrence near the salivary gland tissue, the following three differential diagnoses were considered in our case: 1- low-grade nonintestinal adenocarcinoma, conventional/surface origin type; 2- epithelial-myoepithelial carcinoma; and 3- polymorphous low-grade adenocarcinoma in the salivary gland. Low-grade nonintestinal adenocarcinoma was a possibility owing to the papillary structure of the tumor as well as P63 and CK20 negativity; moreover, CK7 and (occasionally) TTF-1 positivity are also consistent with this type of adenocarcinoma [10, 11]. Although such tumors do not generally present as a solid growth, our patient’s tumor presented as a solid tumor comprising spindle cells. Epithelial-myoepithelial carcinoma also presents as a papillary structure with a solid growth of spindle cells; however, these spindle cells are reportedly positive for SMA, but were negative in our case [12]. Polymorphous low-grade adenocarcinoma presents as a papillary structure with a cribriform pattern and solid growth of cuboidal cells; the proximity of the present case to the salivary gland tissue and its P63 negativity were consistent with these features [13]. However, although S-100 positivity is common, our tumor was negative for this protein. Moreover, TTF-1 positivity in such tumors has not been reported to date.

TTF-1 is a homeodomain-containing tissue-specific transcription factor that belongs to the Nkx2 gene family and is known to play a critical role in cell differentiation and morphogenesis of the thyroid gland and lungs [14]. Antibodies to TTF-1 are widely used in clinical practice for tumors originating from either the thyroid or the lungs [14, 15]. Primary nasopharyngeal papillary adenocarcinoma is microscopically similar to thyroid papillary adenocarcinoma; therefore, an immunohistochemical panel should be used to either determine the subgroup or to exclude the existence of metastatic tumors, especially from the thyroid [7].

Findings such as spindle cells with solid growth, as well as immunostaining analysis that showed negativity for proteins such as thyroglobulin (which is known to be specific only to normal thyroid tissue or its well-differentiated neoplasms) [7], proved useful for our definitive diagnosis. Positive staining for CK7 and CK19, as well as negative staining for CK20, was also informative [2]. Our patient was ultimately diagnosed with biphasic LGNPPA with a prominent spindle cell component [6]. Recently, however, Ozer et al. reported a TL-LGNPPA with focal thyroglobulin expression, and suggested that TL-LGNPPA shares some feature with papillary thyroid carcinoma [16]. Furthermore, they posited that some TL-LGNPPAs may represent papillary thyroid carcinomas originating from an ectopic thyroid tissue or remnants of the nasopharynx [16]. Therefore, in addition to thyroglobulin, we performed immunohistochemical staining for the alternative thyroid transcription factor PAX8 [17, 18]; this protein was not found to be positive in contrast to previously reported tumors.

A previously reported TL-LGNPPA was negative for PAX8 [9] while another was focally positive for thyroglobulin [16]. Moreover, we tested EBER, p16, and HPV because their expression had previously been reported in nasopharyngeal carcinoma [19, 20]; however, our patient’s tumor was negative for these proteins. Hence, much about the histopathology of TL-LGNPPA remains unclear.

TL-LGNPPA is extremely rare; only three reports describing four patients have previously been published with in reference to tumors that express spindle cells (Table 1) [6, 9, 21]. However, the spindle cell component was prominent in only one of these cases. It is unknown whether the etiology of this type of disease is different from that of LGNPPAs that do not exhibit a spindle cell component owing to the small number of cases. Spindle cells can also appear in tissues with squamous differentiation or in morulae. Oide et al. recently reported a patient in which squamous differentiation was observed in a thyroid-like LGNPPA and where immunostaining also showed positivity for 34bE12, CK5/6, p63, and p40 [5]. Only our patient and that of Petersson et al. [6] had tumors exhibiting prominent spindle cells. Furthermore, since the tumors were negative for CK5/6 (data not shown) and p40 (Fig. 4d), we posit that the development of spindle cells occurred independently of squamous differentiation.

**Table 1:**
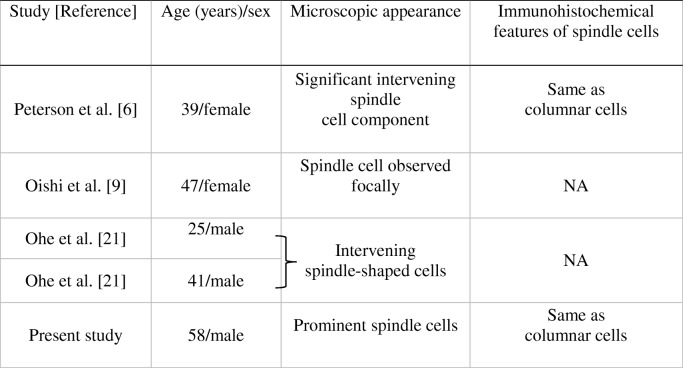
Reported low-grade nasopharyngeal papillary adenocarcinomas with spindle cells

NA: not available

## Conclusion

The implications of positive TTF-1 staining in primary nasopharyngeal papillary adenocarcinoma have previously been described [3–5], but remain largely unclear. The prognosis of TL-LGNPPA is always excellent, with no known incidences of recurrence after complete resection [2, 21]. Therefore, clinicians should be cognizant of this rare disease and perform a proper pathological assessment to avoid unnecessary surgery that could lead to disfigurement. However, long-term monitoring should be performed for any future recurrences should they occur.

## Abbreviations

CT: Computed tomography
EBER: Epstein-Barr-encoded RNA
HPV: Human papillomavirus
LGNPPA: Low-grade nasopharyngeal papillary adenocarcinoma
MRI: Magnetic resonance imaging
SMA: Smooth muscle actin
TL-NPPAC: Thyroid-like nasopharyngeal papillary adenocarcinoma
TTF-1: Thyroid transcription factor-1
